# Differentiation of acute and chronic vertebral compression fractures using conventional CT based on deep transfer learning features and hand-crafted radiomics features

**DOI:** 10.1186/s12891-023-06281-5

**Published:** 2023-03-06

**Authors:** Jun Zhang, Jiayi Liu, Zhipeng Liang, Liang Xia, Weixiao Zhang, Yanfen Xing, Xueli Zhang, Guangyu Tang

**Affiliations:** 1 Department of Radiology, Clinical Medical College of Shanghai Tenth People’s Hospital of Nanjing Medical University, 301 Middle Yanchang Road, Shanghai, 200072 P.R. China; 2 Department of Radiology, Sir RunRun Hospital affiliated to Nanjing Medical University, 109 Longmian Road, Nanjing, Jiangsu 211002 P.R. China; 3 Department of Radiology, Shanghai TenthPeople’s Hospital, Tongji University School of Medicine, 301 Middle Yanchang Road, Shanghai, 200072 P.R. China

**Keywords:** Vertebral compression fracture (VCF), Deep learning, Radiomics, Tomography, X-ray computed, Differential diagnosis

## Abstract

**Background:**

We evaluated the diagnostic efficacy of deep learning radiomics (DLR) and hand-crafted radiomics (HCR) features in differentiating acute and chronic vertebral compression fractures (VCFs).

**Methods:**

A total of 365 patients with VCFs were retrospectively analysed based on their computed tomography (CT) scan data. All patients completed MRI examination within 2 weeks. There were 315 acute VCFs and 205 chronic VCFs. Deep transfer learning (DTL) features and HCR features were extracted from CT images of patients with VCFs using DLR and traditional radiomics, respectively, and feature fusion was performed to establish the least absolute shrinkage and selection operator. The MRI display of vertebral bone marrow oedema was used as the gold standard for acute VCF, and the model performance was evaluated using the receiver operating characteristic (ROC).To separately evaluate the effectiveness of DLR, traditional radiomics and feature fusion in the differential diagnosis of acute and chronic VCFs, we constructed a nomogram based on the clinical baseline data to visualize the classification evaluation. The predictive power of each model was compared using the Delong test, and the clinical value of the nomogram was evaluated using decision curve analysis (DCA).

**Results:**

Fifty DTL features were obtained from DLR, 41 HCR features were obtained from traditional radiomics, and 77 features fusion were obtained after feature screening and fusion of the two. The area under the curve (AUC) of the DLR model in the training cohort and test cohort were 0.992 (95% confidence interval (CI), 0.983-0.999) and 0.871 (95% CI, 0.805-0.938), respectively. While the AUCs of the conventional radiomics model in the training cohort and test cohort were 0.973 (95% CI, 0.955-0.990) and 0.854 (95% CI, 0.773-0.934), respectively. The AUCs of the features fusion model in the training cohort and test cohort were 0.997 (95% CI, 0.994-0.999) and 0.915 (95% CI, 0.855-0.974), respectively. The AUCs of nomogram constructed by the features fusion in combination with clinical baseline data were 0.998 (95% CI, 0.996–0.999) and 0.946 (95% CI, 0.906–0.987) in the training cohort and test cohort, respectively. The Delong test showed that the differences between the features fusion model and the nomogram in the training cohort and the test cohort were not statistically significant (*P* values were 0.794 and 0.668, respectively), and the differences in the other prediction models in the training cohort and the test cohort were statistically significant (*P* < 0.05). DCA showed that the nomogram had high clinical value.

**Conclusion:**

The features fusion model can be used for the differential diagnosis of acute and chronic VCFs, and its differential diagnosis ability is improved when compared with that when either radiomics is used alone. At the same time, the nomogram has a high predictive value for acute and chronic VCFs and can be a potential decision-making tool to assist clinicians, especially when a patient is unable to undergo spinal MRI examination.

**Supplementary Information:**

The online version contains supplementary material available at 10.1186/s12891-023-06281-5.

## Introduction

Vertebral compression fractures (VCFs) are a common, highly disabling injury. The incidence of VCFs continues to increase to the status of a global public health problem that cannot be ignored [1]. Acute or chronic VCFs are important factors that need to be considered when deciding on conservative or surgical treatment [2]. In actual clinical practice, magnetic resonance imaging (MRI) examination plays an important role in the diagnosis of acute fractures. However, its high examination cost, long scanning time, and many contraindications may limit its application in some populations. Computed tomography (CT) examination has limited ability to identify acute fractures, so missed diagnoses may delay treatment. Radiographic indications of acute VCFs are presence of a step defect, presence of a soft-tissue hemorrhage, and a linear white band of condensation [3]. Although these radiographic findings can also be estimated on CT images, Radiologists and orthopedists showed insufficient confidence in evaluating acute VCFs based on CT imaging findings [4], because well-demarcated fracture lines of fresh fractures and sclerotic margins of older fractures are severely hampered by compressed fractures.

Radiomics is a discipline that extracts many quantitative features from medical images and further analyses image features that cannot be observed by the naked eye through advanced algorithmic models [5, 6]. Radiomics is helpful for evaluating the microstructural changes of trabecular bone [7]. Traditional radiomics based on CT images has shown good results in the evaluation of VCFs in the acute and chronic phases and in the identification of benign and malignant VCFs [8, 9]. The diagnosis of acute and chronic VCFs based on CT images by machine learning has also been reported, but it is mainly limited to osteoporotic vertebral compression fractures [10]. Unfortunately, the round region of interest was drawn on the location with highest preserved height of sagittal vertebral body. This may drop the microstructural changes of trabecular bone outside the region of interest. Deep learning mainly uses a filter matrix to perform feature extraction on images through a convolutional neural network (CNN), which requires many labelled datasets to understand the potential relationships between data [11]. Deep transfer learning (DTL) is a process of taking a pre-trained deep learning network [12] and fine-tuning it to learn a new task so that DLR can be applied to a small dataset, a strategy that has become a research hotspot in recent years [13–15]. Therefore, this study aimed to compare DLR combined with traditional radiomics vs. a single radiomics, and to develop and verify the deep learning radiomics nomogram (DLRN) for the differential diagnosis of acute and chronic VCFs.

## Methods

### Patients

**Fig. 1:**
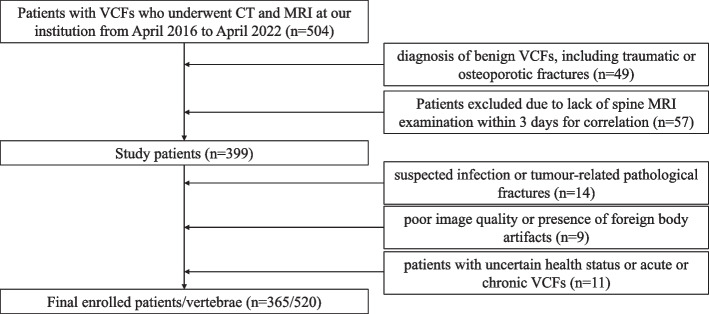
Flow chart of study inclusion

**Fig. 2:**
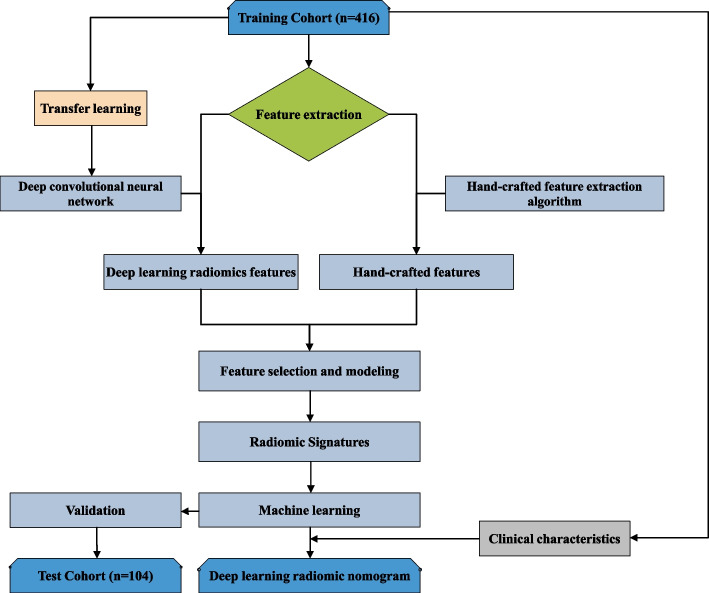
Study flowchart

**Fig. 3:**
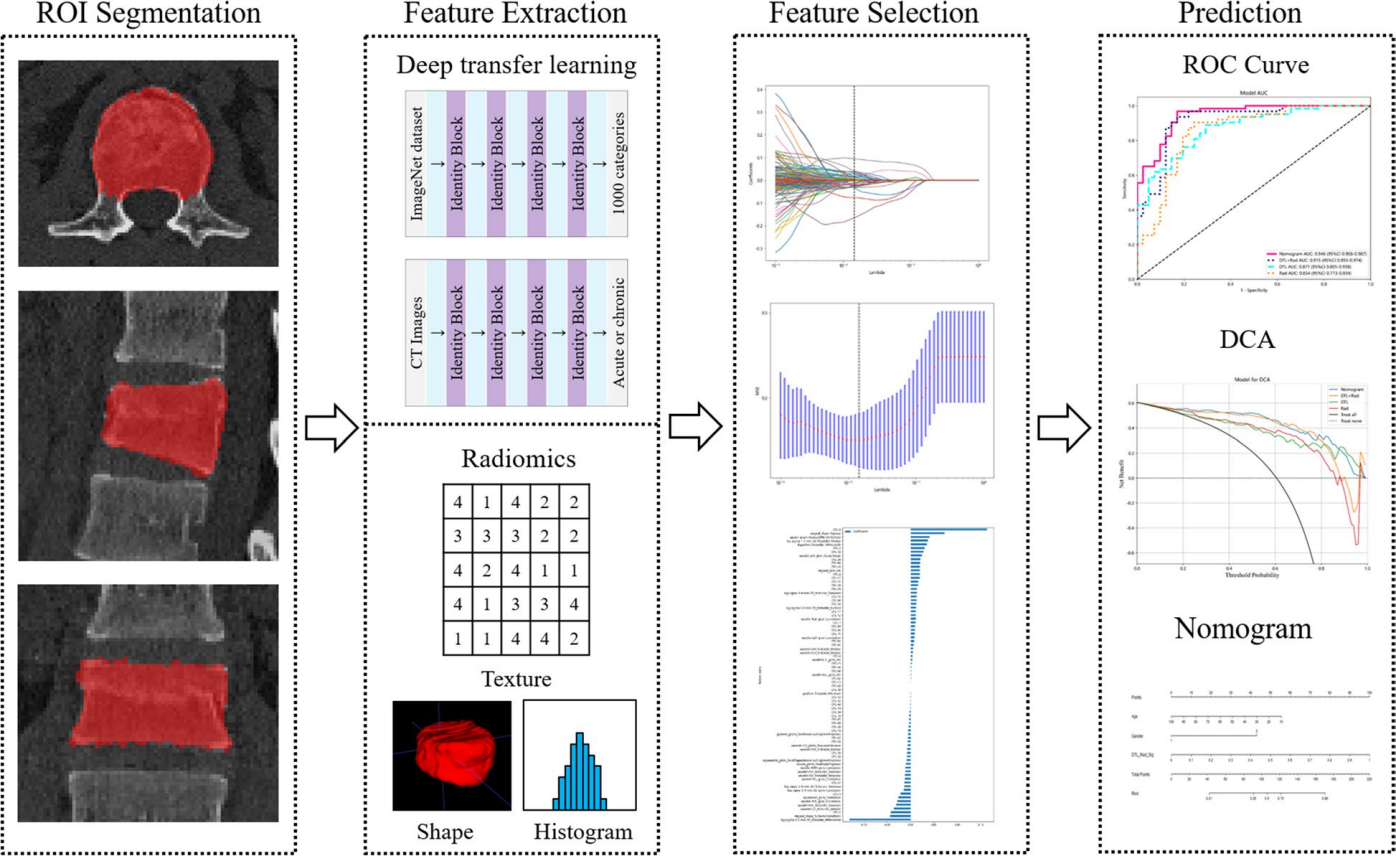
Deep learning radiomics workflow

After review by the hospital’s institutional review committee, the patients were exempted from the requirement to obtain informed consent. We retrospectively analysed the CT and MRI data of 399 patients with thoracic/lumbar compression fractures diagnosed and treated in our hospital between April 2016 and April 2022. The inclusion criteria were as follows: ① diagnosis of benign VCFs, including traumatic or osteoporotic fractures; and ② complete original data, including CT and MRI vertebral examinations, with an interval between the two examinations of less than 3 days. Exclusion criteria: ① suspected infection or tumour-related pathological fractures; ② poor image quality or presence of foreign body artifacts; ③ patients with uncertain health status or acute or chronic VCFs. Acute VCFs was defined as sudden onset of chest and back pain and bone marrow oedema within 4 weeks of MRI examination [16]. The chronic phase was defined as the absence of bone marrow oedema, which was evaluated by two senior doctors with 6 years and 10 years of experience in skeletal and muscle imaging who made a diagnosis of acute or chronic VCFs. When their results are inconsistent, a final conclusion will be reached through their consultation. The detailed screening process is shown in Fig. 1. The flowchart and DLR workflow of this study (Figs. 2 and 3) shows the case collection and grouping, image preprocessing, feature extraction, feature analysis, and model construction. Patients were randomly allocated to training and test cohorts in an 8:2 ratio.

### Clinical baseline characteristics and CT image acquisition

The age and sex of all patients were collected from the clinical medical record system. All CT images were collected with a 256-slice spiral CT scanner (Philips, Brilliance iCT). Scanning parameters were as follows: tube voltage 120 kVp, tube current using an automatic tube current modulation with a fixed noise index. All images (slice thickness: 1.0 mm) were reconstructed using a bone window (width: 1500; window level: 500), then processed and analysed based on the acquired bone window images.

### Image segmentation

**Fig. 4:**
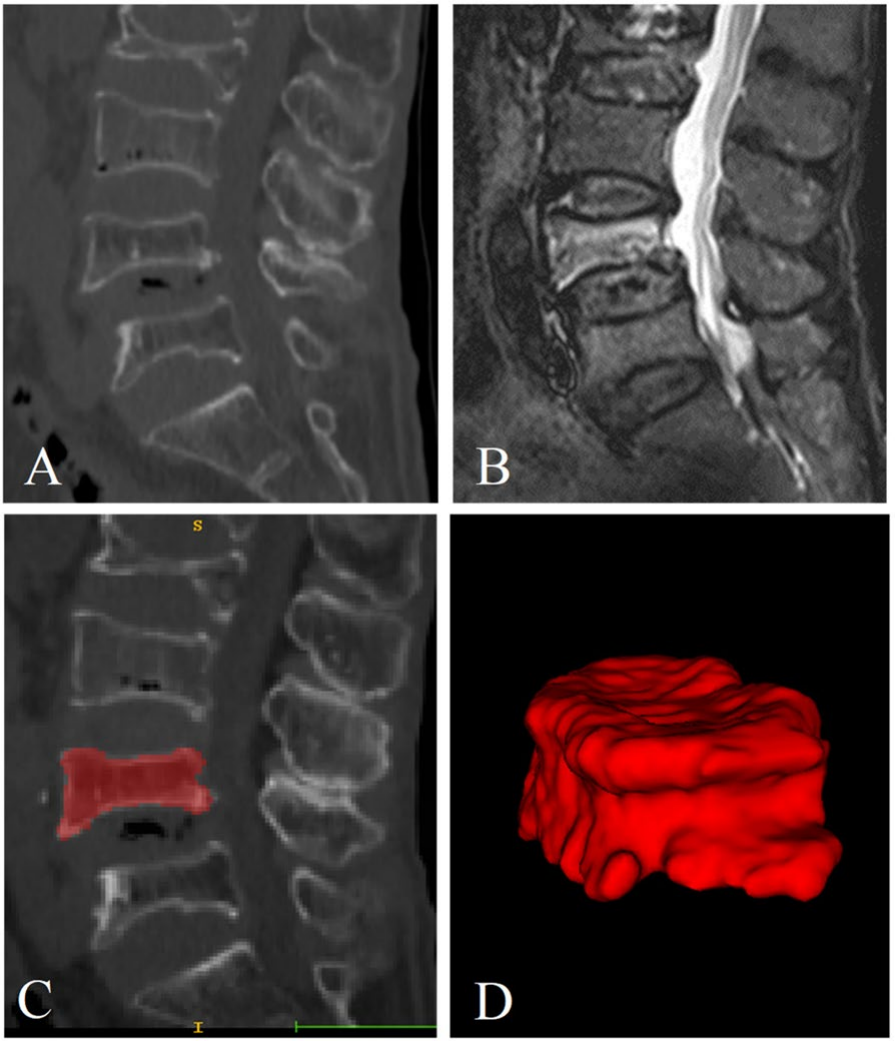
Segmentation of a fractured vertebral body for radiomic analysis in an 82-year-old woman with an acute compression fracture. **A** Compression fracture of L4 on sagittal non-contrast-enhanced spine CT images. **B** Hyperintense on sagittal T2 weighted fat-suppressed imaging of acute vertebral fracture. **C** ROI on sagittal CT images. **D** Three-dimensional volume meshes

Accurate segmentation of fractured vertebrae is a prerequisite for image analysis. Manual segmentation by radiologists was used in this study. First, radiologist A (with 6 years of experience in skeletal and muscle imaging) imported the CT images into ITK-snap software (version 3.8.0, http://www.itksnap.org) and displayed them in three dimensions. The edges of the fractured vertebrae were identified layer by layer and delineated by hand in the sagittal images while avoiding the inclusion of adjacent intervertebral discs, pedicles, or adipose tissues. The fractured vertebral region was completely delineated on each layer of the image and saved as a mask file in “nifti” format after the final cross-sectional and coronal examination (Fig. 4A-D). One month later, 30 patients in the training sequence were randomly selected and re-delineated by radiologist A and radiologist B (with 10 years of experience in skeletal and muscle imaging). Intraclass correlation coefficients (ICCs) were calculated to evaluate the consistency of vertebral delineation within and between observers.

### HCR feature extraction

In order to avoid data leakage, only the training cohort was used in the feature selection part. Before feature extraction, the images were standardized. All images were subjected to isotropic interpolation in advance to generate isotropic 3D data with a pixel spacing of 1 mm, which were unified as the input images for greyscale feature extraction and filtering transformation. The feature extraction algorithm was standardized with reference to the Image Biomarker Standardization Initiative. The open-source software package Pyradiomics (http://pypi.org/project/pyradiomics/) based on the Python 3.6 platform was used to extract the HCR features. The HCR features included first-order features, shape, the greyscale co-occurrence matrix (GLCM), grey-level size zone matrix (GLSZM), grey-level run length matrix (GLLRLM), and neighboring grey-tone difference matrix (NGTDM) and grey level dependence matrix (GLDM). A detailed description of the HCR features extracted in this study can be found in the Pyradiomics document (http://pyradiomics.readthedocs.io).

### DTL feature extraction

**Fig. 5:**
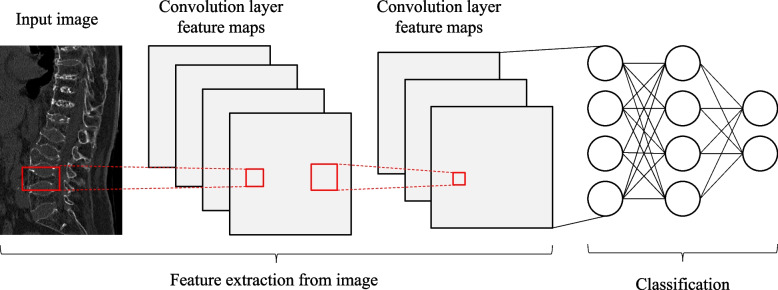
A simple convolutional neural network architecture

**Fig. 6:**
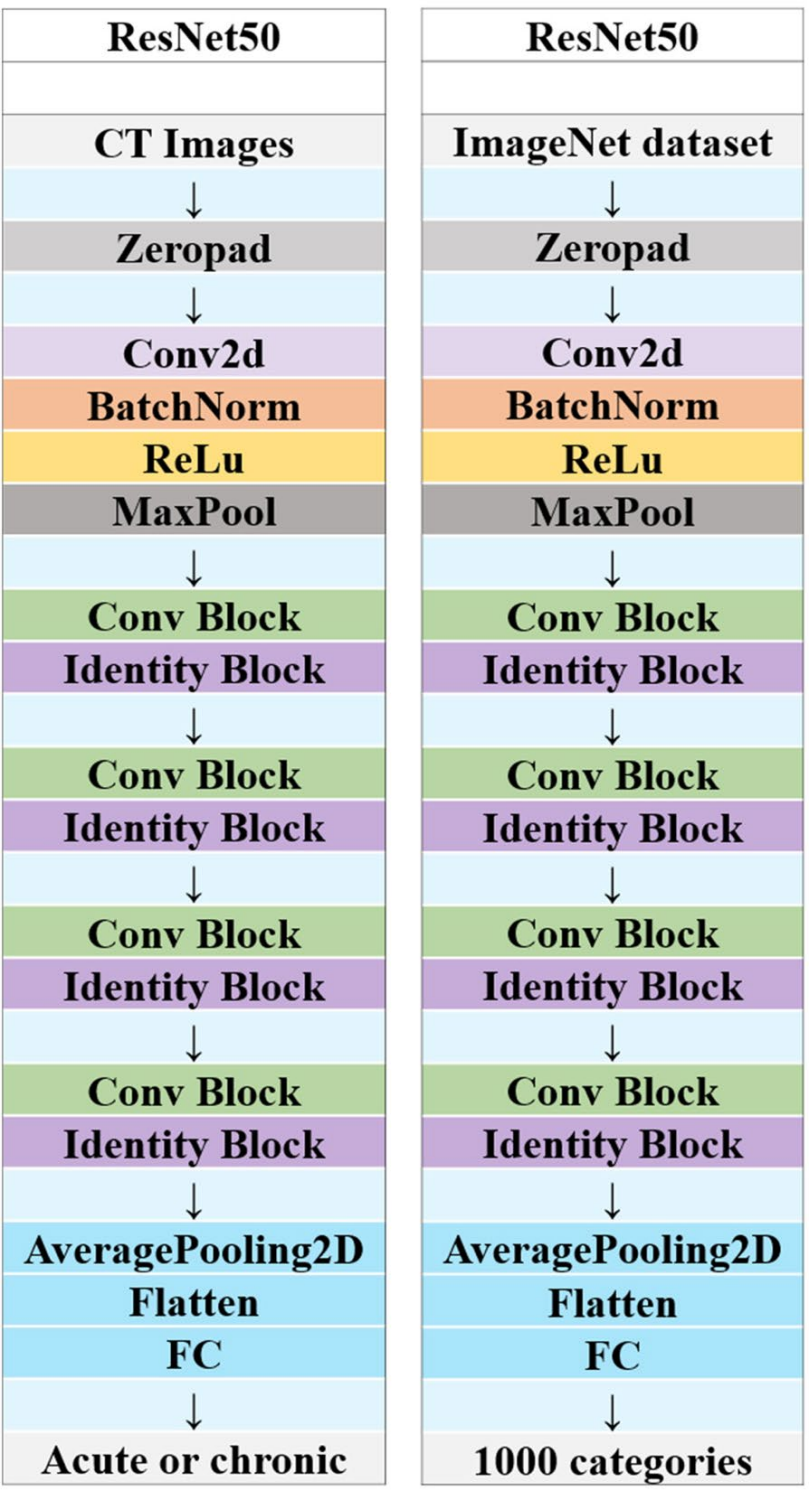
Schematic diagram of the deep convolutional neural network pretraining and fine-tuning network structure

Before extracting the DTL features, the region of interest (ROI) with the largest sagittal area was selected for cropping. The input image is resampled as a 64 × 64 size using linear differences, and the mean and standard deviation of the pixel intensities are normalized to 0 and 1. The image input to the network is a sagittal image, so the input channel is 1. In the deep learning library PyTorch based on the Python 3.6 platform [17], DTL similar to earlier methods [18, 19] was applied. We chose ResNet50 (Figs. 5 and 6) as the basic model of our transfer learning model, and we carefully set the learning rate to better perform transfer.

Since the second-to-last layer of the model (AveragePooling layer) was selected as the transfer feature, we divided the model parameters into two parts: 1. backbone and 2. task-spec. The parameters of the task-spec part were randomly initialized. The initialization of the backbone part used the model parameters pretrained by ImageNet. For the parameters of the task-spec part, we used the cosine annealing learning rate decay algorithm for reference [20]. It varies with the number of iterations is shown in Supplementary 1.

### Feature selection and fusion

To screen the HCR features with good reproducibility and low redundancy, the ICCs between the HCR features were first calculated. The HCR features with ICC ≥ 0.8 were selected twice [21], and the feature quantity decreased from 1648 to 1589. Second, for the features with high repeatability, Spearman’s rank correlation coefficient was calculated to express the relationships between the features, and one of any pair of features with a correlation coefficient greater than 0.9 was retained. To preserve the characterization ability of features to the greatest extent, we used the greedy algorithm for feature selection; that is, the feature with the highest redundancy in the current set was deleted each time. After Spearman correlation coefficient screening and greedy selection, the number of features decreased from 1589 to 224. Finally, using the least absolute shrinkage and selection operator (LASSO) algorithm, we shrank some regression coefficients by constructing a penalty function λ to force them to become 0, thereby incorporating the stable HCR features into LASSO-Cox analysis. Ten-fold cross-validation was performed to determine the optimal λ value based on the minimum value standard. According to the model corresponding to the optimal λ value, we screened for the radiomics parameters with nonzero coefficients and their weights. Correlation analysis was performed on the relevant features selected by the LASSO-Cox algorithm to eliminate redundancy, and the parameters with correlation coefficients greater than 0.5 were removed. Finally, the independent and stable HCR features were retained.

Since the dimension of deep transfer features is 2048, to ensure balance between features, we used principal component analysis to reduce the dimensionality of deep transfer learning features and reduce the deep learning model to 100 dimensions to improve the generalization ability of the model and to reduce the risk of overfitting.

After fusing the selected hand-crafted features and deep learning features, the Z score method was used to standardize all the features fusion, and the mean and variance of each column of features were calculated. Each column of features was converted into a standard normal distribution by subtracting the mean and dividing by the variance. In the feature fusion stage, we performed early fusion of the features screened by the hand-crafted method and the deep transfer features to form a complete feature set. Finally, LASSO-Cox was used to screen out the features with a coefficient of nonzero, and the features fusion were selected and dimensionality-reduced to find the subset with optimal feature fusion. Finally, a 74-dimensional feature fusion subset was obtained.

### Models construction and validation

After feature fusion and screening, we used the scikit-learn machine learning library to construct a machine learning classification model. The machine learning classification model included a support vector machine (SVM), k-nearest neighbour (KNN), decision trees, random forest (RF), extremely randomized trees, eXtreme gradient boosting (XGBoost), and light gradient boosting machine. All models were trained by using grid search algorithm in training cohort. Common used parameters in each model was considered to be tuned. The performance of different classification models was compared. To prevent overfitting, 5-fold cross-validation was done to select the optimal parameters for the classification model in the training sequence. Finally, the optimal Radiomics feature importance score was obtained. The receiver operating characteristic curve (ROC) was plotted, and the area under the curve (AUC), accuracy, sensitivity, and specificity were calculated to evaluate the performance of various prediction models. The DLRN of feature fusion model combined with Clinical baseline characteristics was drawn to visualize the classification assessment.

### Statistical analysis

All statistical tests were performed using R software version 4.0.2. Delong test was used to compare the AUC of various prediction models, and Decision curve analysis (DCA) was used to compare the clinical values of various prediction models. The nomogram and DCA were calculated mainly by using R “rmc” and “rmda” packages. The statistical significance for all two-sided tests was set at *P* < 0.05.

## Result

### Clinical baseline characteristics

Among the 399 patients who met the inclusion criteria, 34 were excluded because of ① infection-related fractures (14 cases), ② poor image quality or the presence of foreign body artifacts (nine cases), and ③ uncertain poor health status or fracture staging (11 cases). Finally, 365 patients were included in the study, including 149 males and 216 females, with an age range of 17 to 95 years and an average age of 61.7 ± 17.0 years. Among them were 315 acute stage VCFs (thoracic vertebrae: 111; lumbar vertebrae: 204; patients aged 17-95 years, with an average age of 59.3 ± 18.1 years) and 205 chronic stage VCFs (thoracic vertebra: 88; lumbar vertebra: 117; patients aged 39-95 years, with an average age of 71.1 ± 11.9 years). The baseline characteristics of the acute and chronic VCFs are summarized in Table 1.

**Table 1:** Baseline Patient Characteristics in the Acute and Chronic VCFs

	Acute VCFs (*n* = 253)	Chronic VCFs (*n* = 112)	*p*-value
Age (years)^a^	59.3 ± 18.1 (17-95)	71.1 ± 11.9 (39-95)	0.225
Females/Males	164/89	78/34	0.812
Number of VCFs (thoracic, lumbar)	315 (111, 204)	205 (88,117)	0.956^#^

^#^*p*-value for difference in the number of acute and chronic fractures

^a^ Data are mean ± standard deviation (range) age at the time of CT examination

### Features selection

**Fig. 7:**
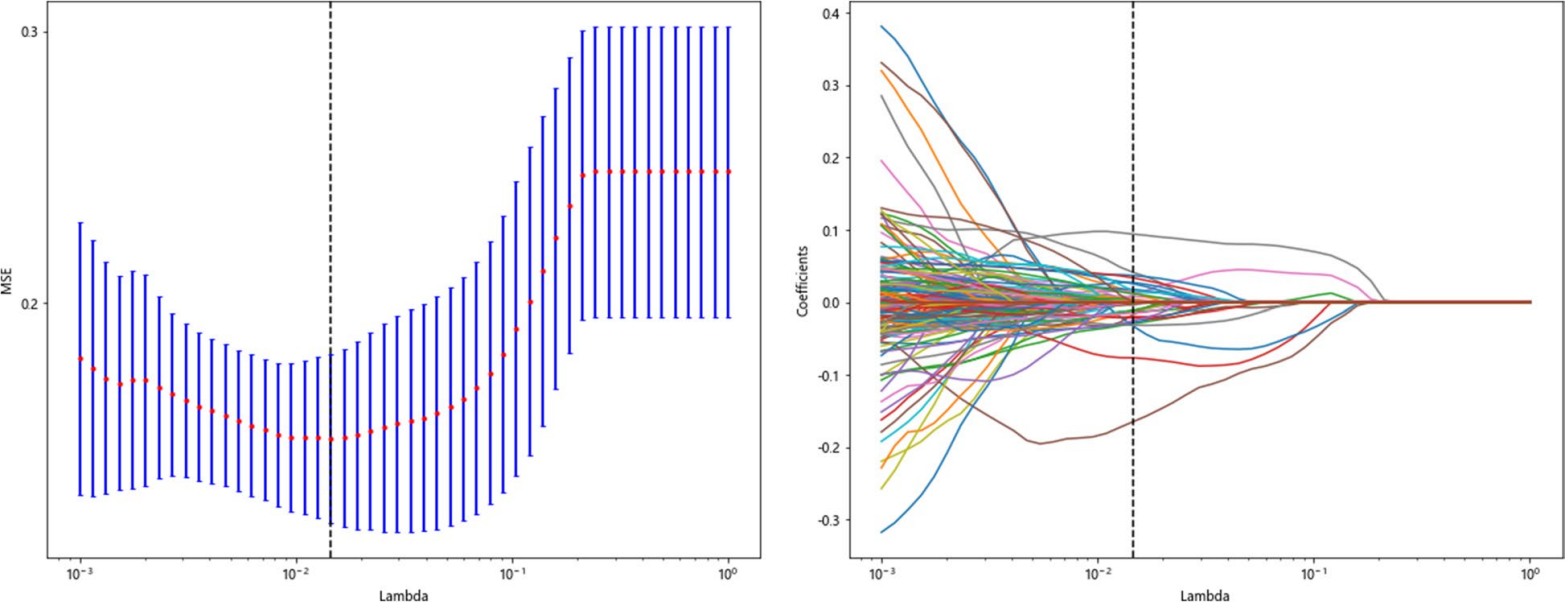
Hand-crafted feature selection using the least absolute shrinkage and the histogram of the Radiomics feature importance score based on the selected features. The optimal λ value of 0.014 was selected

LASSO-Cox regression analysis was used to perform the dimensionality reduction of the HCR. The selection of the penalty coefficient (λ = 0.014), the process of feature selection, and the curve of the variation of the feature coefficient with λ are shown in (Fig. 7). After the final screening, a total of 41 HCR were selected, including first-order statistical features (19), shape features (2), GLCM (15), GLSZM (4) and GLRLM (1). The Radiomics feature importance score was constructed from the 40 HCR and their corresponding regression coefficients. The Radiomics feature importance score formula is shown in Supplementary 2.

**Fig. 8:**
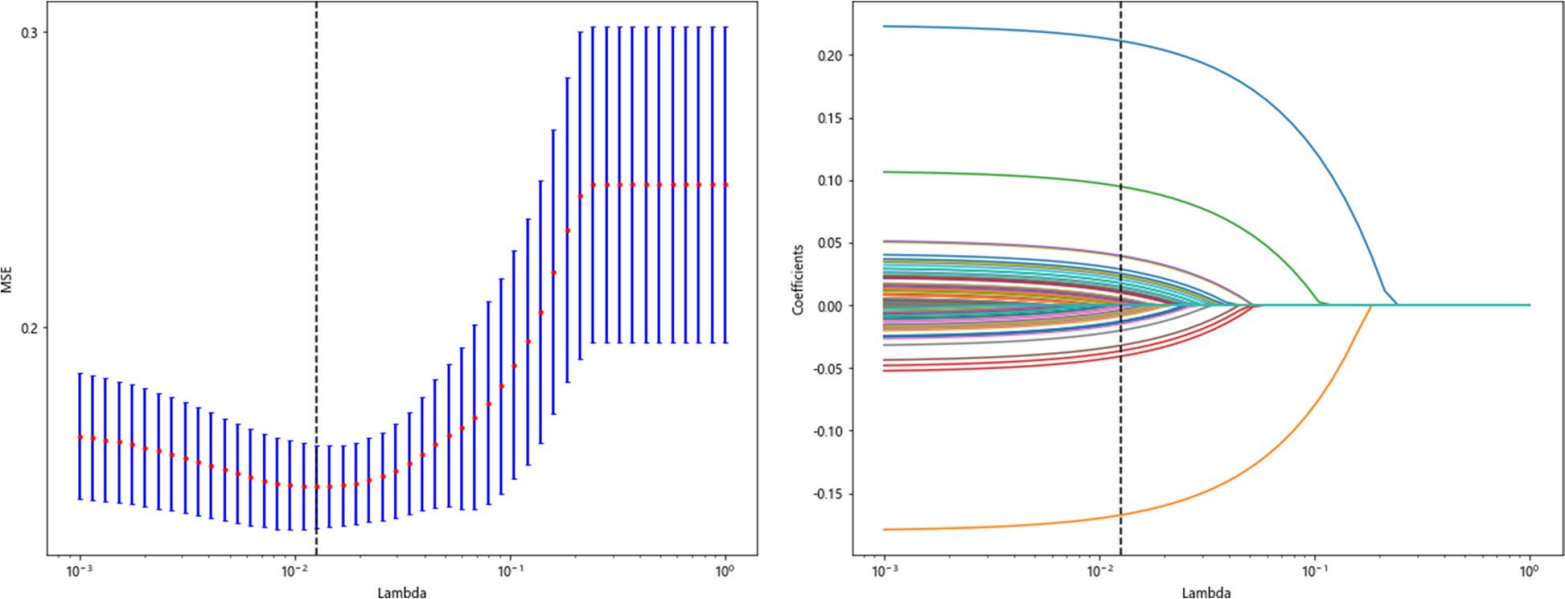
DTL feature selection using the least absolute shrinkage and the histogram of the Deep Learning feature importance score based on the selected features. The optimal λ value of 0.012 was selected

LASSO-Cox regression analysis was used to perform the dimensionality reduction of DTL features. The selection of the penalty coefficient (λ = 0.012), the process of feature selection, and the curve of the variation of the feature coefficient with λ are shown in (Fig. 8). After the final screening, a total of 50 DTL features were selected. The Deep Learning feature importance score was constructed from the 50 DTL features and their corresponding regression coefficients. The Deep Learning feature importance score formula is shown in Supplementary 3.

**Fig. 9:**
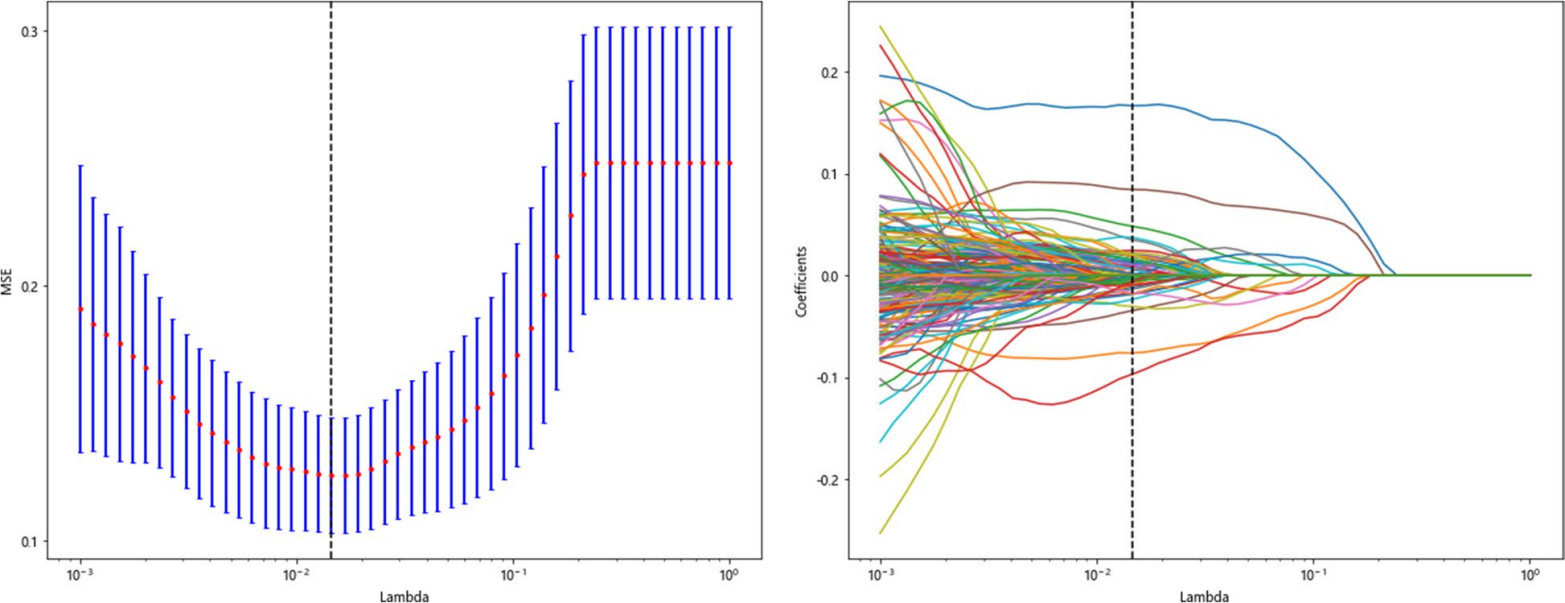
Fusion feature selection using the least absolute shrinkage and the histogram of the Deep Learning Radiomics feature importance score based on the selected features. The optimal λ value of 0.014 was selected

LASSO Cox regression was used to perform dimensionality reduction of features fusion. The selection of the penalty coefficient (λ = 0.014), the feature screening process, and the graph of the variation of the feature coefficient with λ are shown in (Fig. 9). After the final screening of the features fusion, 30 hand-crafted features and 44 DTL features were retained, and the Deep Learning Radiomics feature importance score was constructed from the features fusion and their corresponding regression coefficients. The Deep Learning Radiomics feature importance score formula is given in Supplementary 4.

### Predictive performance of the models

**Fig. 10:**
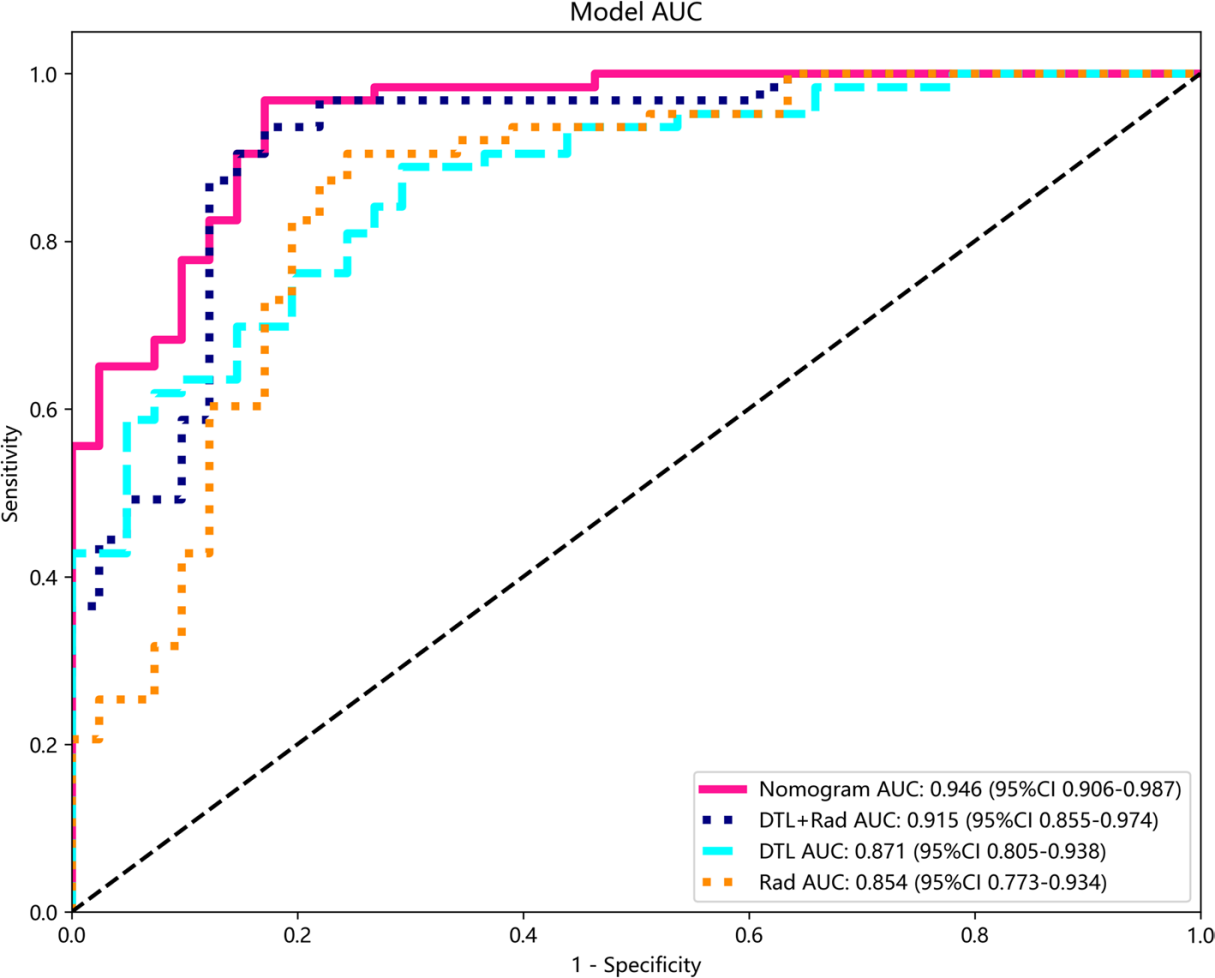
The AUCs of various prediction models in the test cohort

**Fig. 11:**
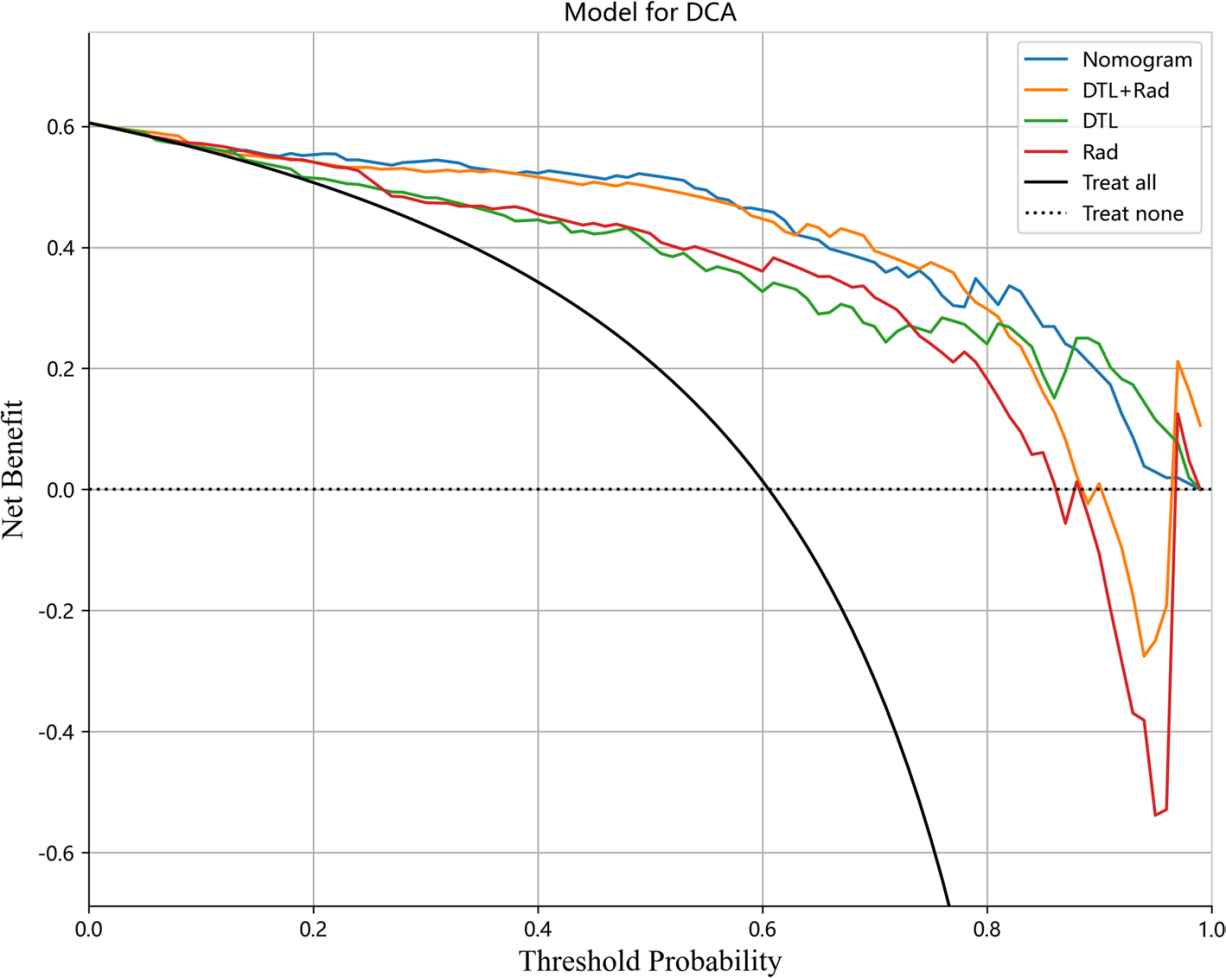
Decision curve analysis was developed with various prediction models

SVMs are the most effective machine learning algorithms in DLR, traditional radiomics, and the prediction model of their fusion (Supplementary 5). As shown in Table 2 and Fig. 10, the optimal prediction model is the features fusion, with an AUC of 0.997 (95% CI, 0.994–0.999) for the training cohort and 0.915 (95% CI, 0.855–0.974) for the test cohort. The features fusion were combined with the clinical baseline data to construct a nomogram with an AUC of 0.998 (95% CI, 0.996–0.999) for the training cohort and 0.946 (95% CI, 0.906–0.987) for the test cohort. Using the Delong test, there was no significant difference between the features fusion model and nomogram in the training cohort and the test cohort (*P* values were 0.794 and 0.668, respectively), and the differences in the other prediction models between the training cohort and the test cohort were statistically significant (*P* < 0.05). DCA demonstrated that the nomogram was more beneficial to the patient than the DLR, traditional radiomics, and features fusion prediction models (Fig. 11).

**Table 2:** Diagnostic efficiency of different models in the training cohort and test cohort

Model	Training cohort					Test cohort				
	AUC (95% CI)	ACC (%)	SEN (%)	SPE (%)	F1-score	AUC (95% CI)	ACC (%)	SEN (%)	SPE (%)	F1-score
Radiomics	0.973 (0.955-0.990)	92.3	95.2	92.0	0.950	0.854 (0.773-0.934)	84.6	77.8	75.1	0.802
DLR	0.992 (0.983-0.999)	94.9	94.4	96.1	0.958	0.871 (0.805-0.938)	81.7	88.9	70.7	0.855
Features Fusion	0.997 (0.994-0.999)	97.1	96.0	98.1	0.973	0.915 (0.855-0.974)	88.5	93.6	82.9	0.914
DLRN	0.998 (0.996-0.999)	98.2	95.3	97.0	0.975	0.946 (0.906-0.987)	90.1	92.6	85.2	0.925

*ACC* Accuracy, *SEN* Sensitivity, *SPE* Specificity

**Fig. 12:**
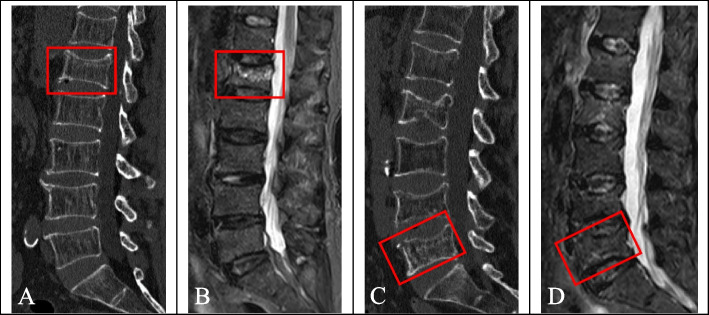
Cases correctly diagnosed by all the prediction models. Figs. **A** and **B** show a case correctly diagnosed as acute fracture (male, 87 years old, history of minor trauma, L1 acute vertebral fracture). Figs. **C** and **D** show a case correctly diagnosed as chronic fracture (female, 61 years old, with no history of trauma, with multiple acute and chronic fractures of the lumbar spine)

**Fig. 13:**
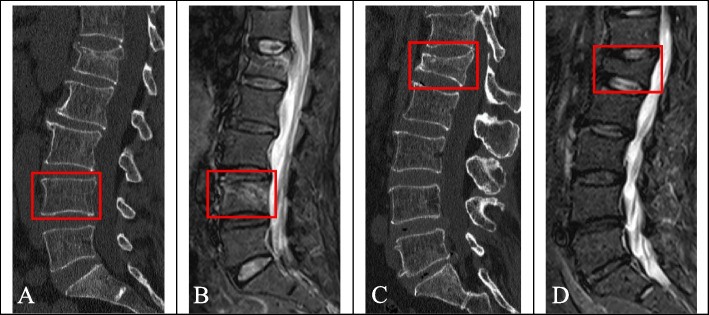
Cases incorrectly diagnosed by the doctor but correctly diagnosed by all the predictive models. Figs. **A** and **B** show a case of acute fracture that was misdiagnosed by the doctor (female, 57 years old, history of traffic accident and trauma, and of the L4 acute vertebral fracture). Figs. **C** and **D** show a case of chronic fracture mistakenly diagnosed by the doctor (female, 63 years old, history of minor trauma, L1 chronic vertebral fracture)

Figure 12A-D shows the cases with acute or chronic fractures correctly diagnosed by all the prediction models. The presence of a condensation band was a feature of acute fractures, and chronic fractures generally only had wedge-shaped or biconcave changes. When the characteristics of acute and chronic fractures are not typical, the diagnosis may be erroneous, depending on the diagnosis experience of the doctor. Figure 13A-D shows the cases erroneously diagnosed by the doctor but correctly diagnosed by all the prediction models, indicating that abnormal manifestations of a few acute fractures that were visible to the naked eye did not appear on CT images, and the presence of condensation band was also found in a few chronic fractures.

### Construction of the deep learning radiomics nomogram

**Fig. 14:**
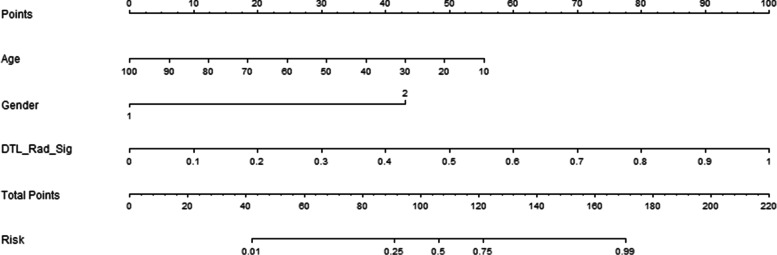
Deep learning radiomic nomogram was developed with the age, gender and feature fusion

The comparison of three prediction models for acute and chronic VCFs showed that the feature fusion prediction model had good prediction performance and great clinical benefit. The use of features fusion combined with Clinical baseline characteristics to construct a nomogram can be used to visually distinguish between acute and chronic VCFs (Fig. 14).

## Discussion

VCFs are often accompanied by acute or recurrent thoracic and lumbar pain. Some patients may develop VCFs based on chronic compression fractures, so imaging usually shows multiple fractures in different stages [22–24]. In clinical work, MRI is mainly used to display bone marrow oedema to judge acute fracture. However, factors such as high examination cost, long scanning time, and more contraindications, especially the lack of MRI medical equipment in primary hospitals, may delay the timely formulation of treatment plans for VCFs. In recent years, Petritsch et al. [25] have used dual-energy CT virtual noncalcium (VNCa) technique to evaluate bone marrow oedema in traumatic VCFs and showed good diagnostic performance (AUC = 0.922). Some scholars also propose that 5 CT findings, including cortical or endplate fracture line, trabecular fracture line, condensation band, change in trabecular attenuation, and width of paravertebral soft-tissue change, are closely related to the presence and extent of bone marrow oedema [26]. However, in actual work, grassroots hospitals still do not have high-end CT with dual-energy CT technology. It is difficult to effectively promote the prediction of bone marrow oedema based on the VNCa technique for grassroots hospitals. Therefore, developing an appropriate and convenient method for the timely diagnosis of acute and chronic VCFs is the key scientific problem to be solved in this study. First, the ability of DLR to differentiate between acute and chronic VCFs was compared with that of radiomics. Subsequently, a features fusion model based on DLR in combination with radiomics and a nomogram was established to assess the potential diagnostic value in differentiating acute and chronic VCFs as a potential decision-making tool for clinicians, especially when spine MRI is not available to the patient.

Radiomics is a technology developed in recent years that can extract a large number of HCR features to improve the ability of diagnosis and prognostic prediction, and HCR features need to be specified by a human in advance [27]. Yang et al. [10] used a data set of 147 patients, which were assigned to a training cohort (acute fractures: 46, chronic fractures: 57) and a validation cohort (acute fractures: 26, chronic fractures: 18). And they constructed 14 HCR features based on CT images to distinguish acute and chronic osteoporosis VCFs, and they showed good diagnostic efficiency (the AUC of the test cohort was 0.82). In this study, 41 HCR features were used to construct the prediction model, and the test cohort AUC was 0.854, which was comparable with the results of Yang et al. (AUC, 0.82). Yang’s selection of the sagittal maximum area level of the vertebral body to delineate a two-dimensional circular ROI is related to the fact that it may miss features information such as cortical or endplate fractures. We selected three-dimensional of the vertebral body as ROI so that it fully represented the features of the entire vertebral body. Kim et al. [28] used a total of 238 fractures (159 acute and 79 chronic) in 122 patients and 58 fractures (39 acute and 19 chronic) in 32 patients were included in the training and test cohorts respectively. The AUC of the HCR features and CT findings was 0.95 in the training cohorts and 0.93 in the test cohorts. In this study, to ensure the robustness and generalization ability of the prediction model, we have read a large number of literature and recorded the sample size of deep learning and radiomic. In addition, during the preliminary experiment of this study, a variety of radiomic feature calculation methods were adopted, as well as a variety of methods for screening relatively stable features from deep learning features. Also, deep transfer learning is an effective way to solve the insufficiency of the dataset, of which fine-tuning is a common method. Finally, we selected 520 cases of VCFs to be included in the group and have used fine-tuning. In our study, a total of 520 VCFs (315 acute and 205 chronic) were assigned to a training cohort (acute fractures: 252, chronic fractures: 164) and a validation cohort (acute fractures: 63, chronic fractures: 41). The AUCs of the features fusion model in the training cohort and test cohort were 0.997 and 0.915, which was comparable with the results of Kim et al.

CNNs are deep learning models widely used in the field of computer imaging and vision. Only when trained on large enough datasets can CNNs correctly learn its features, but it is difficult to obtain such datasets in clinical practice [29]. In the initial study, we tried to use a variety of convolutional neural networks, such as ResNet, VGGnet and so on. Compared with other CNN models, ResNet’s structure uses shortcut connection, which can effectively realize fusion training. Moreover, ResNet’s classic network structures include ResNet-18, ResNet-34, ResNet-50, ResNet-101, and ResNet-152. ResNet-18 and ResNet-34 have the same basic structure and belong to relatively shallow networks. The basic structures of the latter three are different from those of ResNet-18 and ResNet-34, and belong to deeper networks. Because of the deep network structure of ResNet101 and ResNet152, many model parameters also bring some difficulties to the training data, which leads to their performance degradation. So we finally chose ResNet50, which is generally recognized by the public. Now DLR methods have mostly been used for tumour classification and prognosis [30–32], while there is little application in other fields, such as skeletal system. In our study, it was found that the differential diagnosis ability of the feature fusion model was improved compared with that of using either radiomics alone. Finally, we found that the DLR had high predictive value for acute and chronic VCFs, with AUCs of 0.998 and 0.946 in the training cohort and test cohort, respectively. Although there was no significant difference in the AUCs between the nomogram and feature fusion model in the training cohort and test cohort, DCA showed that the nomogram brought more benefits to patients. Also, the HCR and DTL features were extracted from conventional CT images. To our knowledge, there is no report on integrating HCR and DTL features for distinguishing acute and chronic VCFs. Besides, our research is based on some ordinary image data and does not require special training, so it has significant potential. Although the interpretability of the current deep migration learning features needs to be further studied, it does not preclude the mapping of the features of the lesion itself in the convolution operation, so that it can be further used for the construction and classification of the prediction model.

In our study, 30 radiomic features and 44 deep transfer learning features were finally screened to construct a fusion feature prediction model. As shown in Fig. 12, the correlation coefficient of shape_Flatness is higher. Flatness shows the relationship between the largest and smallest principal components in the ROI shape. The distinguishing point of acute and chronic fracture is bone marrow edema. The histological features in bone marrow oedema were characterized by hematoma and inflammatory exudative edema. The corresponding densities of BME presented a diffuse and uniform morphological distribution, Chronic VCFs has no bone marrow edema [33]. Therefore, the edge and morphological characteristics of them are different. GLSZM quantifies gray level zones in an image. A gray level zone is defined as the number of connected voxels that share the same gray level intensity. The correlation coefficient of Zone Variance is higher. Zone Variance measures the variance in zone size volumes for the zones. Zone Variance reflects the heterogeneity of Hepatocellular carcinoma [34], and one possible explanation is that acute VCFs are considered to be associated with increased bone marrow water content, while chronic VCFs fracture healing results in the generation of large amounts of freshly woven bone with less water content. First-order statistics describes the distribution of voxel intensity within the image region defined by the ROI through common basic indicators. The correlation coefficient of First order_10 percent is high. It had a high positive correlation with the CT value of vertebral and osteoporosis [35, 36]. This is supported by the fact that about half of the cases in our study were osteoporotic fractures.

There are some limitations to this study that can be further studied and explored in future work. First, the sample size of VCFs was small. Although our findings reflect the predictive ability of deep learning features to a certain extent, more data would be more convincing, so multicentric studies should be conducted to expand the dataset. Second, this was a retrospective study. In the future, more prospective data are needed to verify the effectiveness of the model. Finally, the deep learning features extracted by the DLR method are hard for humans to interpret [37, 38]. Therefore, future studies on the interpretability of imaging features will help to further enhance the value of DLR in clinical application.

## Conclusions

In conclusion, the DLR features were fused with the HCR features based on CT images, which improved the identification ability of a single radiomics prediction model for acute and chronic VCFs, especially when a patient is unable to undergo spinal MRI examination.

## Supplementary Information

**Additional file 1.**

**Additional file 2.**

**Additional file 3.**

**Additional file 4.**

**Additional file 5.**

## Data Availability

The datasets generated and/or analysed during current study are not publicly available as CT data and DICOM headers contain patient information. But they are available from the corresponding author on reasonable request.

## Abbreviations

DLR: Deep learning radiomics
HCR: Hand-crafted features radiomics
VCFs: Vertebral compression fractures
CT: Computed tomography
DTL: Deep transfer learning
ROC: Receiver operating characteristic
DCA: Decision curve analysis
CI: Confidence interval
MRI: Magnetic resonance imaging
CNN: Convolutional neural network
DLRN: Deep learning radiomics nomogram
ICCs: Intraclass correlation coefficients
GLCM: Greyscale co-occurrence matrix
GLSZM: Grey-level size zone matrix
GLLRLM: Grey-level run length matrix
NGTDM: Neighboring grey-tone difference matrix
GLDM: Grey level dependence matrix
ROI: Region of interest
LASSO: Least absolute shrinkage and selection operator
SVM: Support vector machine
KNN: K-nearest neighbor
RF: Random forest
XGBoost: eXtreme gradient boosting
VNCa: Virtual noncalcium
