# Supplementary material for: Differentiation of acute and chronic vertebral compression fractures using conventional CT based on deep transfer learning features and hand-crafted radiomics features
Source: BMC Musculoskelet Disord. 2023 Mar 6;24:165. doi: 10.1186/s12891-023-06281-5 (PMC9987077; doi:10.1186/s12891-023-06281-5)
Supplement: Supplementary file 1 — Additional file 1. [file 12891_2023_6281_MOESM1_ESM.docx]

$$\eta_{t}^{task-spec}=\eta_{\min}^{i}+\frac{1}{2}(\eta_{\max}^{i}-\eta_{\min}^{i})(1+\cos(\frac{T_{cur}}{T_{i}}\pi))$$

Where$\eta_{\min}^{i}=0$,$\eta_{\max}^{i}=0.01$,$T_{i}=30$represent the minimum learning rate, the maximum learning rate, and the number of iterative epochs, respectively. Because the backbone part uses pretraining parameters, to ensure the transfer effect, we have

$$T_{cur}=\frac{1}{2}T_{i}$$

The parameters of the backbone part were fine-tuned. Therefore, the learning rate of the backbone part was as follows:

$$\eta_{t}^{backbone}=\left\{\begin{matrix} \begin{matrix} 0 & & & & & & & & & & & T_{cur}\leq\frac{1}{2}T_{i} \end{matrix} \\ \begin{matrix} \eta_{\min}^{i}+\frac{1}{2}(\eta_{\max}^{i}-\eta_{\min}^{i})(1+\cos(\frac{T_{cur}}{T_{i}}\pi)) & T_{cur}>\frac{1}{2}T_{i} \end{matrix} \end{matrix}\right.$$
