# Supplementary material for: Differentiation of acute and chronic vertebral compression fractures using conventional CT based on deep transfer learning features and hand-crafted radiomics features
Source: BMC Musculoskelet Disord. 2023 Mar 6;24:165. doi: 10.1186/s12891-023-06281-5 (PMC9987077; doi:10.1186/s12891-023-06281-5)
Supplement: Supplementary file 2 — Additional file 2. [file 12891_2023_6281_MOESM2_ESM.docx]

Radiomics feature importance score = 0.6021054863381694 -0.003179 * exponential_glcm_Correlation +0.018784 * exponential_glcm_Imc1 +0.001782 * exponential_glrlm_GrayLevelNonUniformity +0.005964 * gradient_glcm_Idn +0.021938 * log-sigma-1-0-mm-3D_firstorder_Median -0.025760 * log-sigma-3-0-mm-3D_firstorder_Skewness +0.017453 * log-sigma-3-0-mm-3D_glszm_ZoneVariance -0.157097 * log-sigma-5-0-mm-3D_firstorder_90Percentile +0.011138 * log-sigma-5-0-mm-3D_firstorder_Kurtosis -0.031281 * log-sigma-5-0-mm-3D_glcm_Correlation +0.024243 * logarithm_firstorder_10Percentile -0.006875 * logarithm_glcm_Correlation +0.000208 * logarithm_glcm_Idn +0.084818 * original_shape_Flatness -0.106971 * original_shape_SurfaceVolumeRatio +0.013114 * square_firstorder_Range -0.000794 * square_glcm_Correlation +0.010578 * square_glcm_Idn +0.029775 * square_glszm_SmallAreaLowGrayLevelEmphasis -0.026556 * squareroot_glcm_Correlation +0.001572 * squareroot_glszm_SmallAreaHighGrayLevelEmphasis +0.005100 * wavelet-HHH_firstorder_Mean -0.025734 * wavelet-HHL_firstorder_Median -0.008784 * wavelet-HHL_firstorder_Skewness -0.026188 * wavelet-HHL_glcm_Correlation +0.002317 * wavelet-HLH_firstorder_Maximum +0.030084 * wavelet-HLH_firstorder_Median -0.023088 * wavelet-HLH_firstorder_Skewness +0.037334 * wavelet-HLH_glcm_Correlation +0.026826 * wavelet-HLL_firstorder_Mean -0.014621 * wavelet-HLL_firstorder_Skewness -0.021573 * wavelet-HLL_glcm_Correlation +0.011219 * wavelet-LHH_firstorder_Median -0.000869 * wavelet-LHH_glcm_Idn -0.004246 * wavelet-LHL_firstorder_Mean -0.024829 * wavelet-LHL_firstorder_Median -0.001249 * wavelet-LHL_firstorder_Skewness -0.004214 * wavelet-LHL_glcm_ClusterShade +0.013426 * wavelet-LHL_glszm_SmallAreaHighGrayLevelEmphasis -0.027122 * wavelet-LLH_firstorder_Median +0.016913 * wavelet-LLH_glcm_ClusterShade
