# Supplementary material for: Differentiation of acute and chronic vertebral compression fractures using conventional CT based on deep transfer learning features and hand-crafted radiomics features
Source: BMC Musculoskelet Disord. 2023 Mar 6;24:165. doi: 10.1186/s12891-023-06281-5 (PMC9987077; doi:10.1186/s12891-023-06281-5)
Supplement: Supplementary file 3 — Additional file 3. [file 12891_2023_6281_MOESM3_ESM.docx]

Deep Learning feature importance score = 0.5992600310821189 + +0.195290 * DTL-0 -0.191805 * DTL-1 +0.100085 * DTL-2 -0.036235 * DTL-3 -0.043316 * DTL-5 +0.038013 * DTL-8 +0.019155 * DTL-10 -0.015424 * DTL-11 +0.042674 * DTL-12 +0.004483 * DTL-15 +0.029200 * DTL-17 +0.009320 * DTL-18 -0.010574 * DTL-20 -0.021037 * DTL-23 +0.044176 * DTL-24 -0.003129 * DTL-26 -0.029027 * DTL-27 -0.010190 * DTL-28 +0.022127 * DTL-29 +0.025924 * DTL-31 -0.005355 * DTL-34 -0.007041 * DTL-36 +0.019993 * DTL-37 +0.005819 * DTL-38 -0.022215 * DTL-40 +0.012085 * DTL-42 -0.057792 * DTL-43 +0.013254 * DTL-45 -0.004983 * DTL-47 -0.008056 * DTL-52 +0.009586 * DTL-54 -0.001913 * DTL-55 +0.007106 * DTL-56 +0.002807 * DTL-57 -0.010231 * DTL-58 -0.003221 * DTL-62 +0.018374 * DTL-69 -0.022176 * DTL-70 +0.000610 * DTL-71 -0.003283 * DTL-76 +0.010408 * DTL-77 +0.018172 * DTL-80 -0.003743 * DTL-81 -0.004629 * DTL-85 +0.002229 * DTL-87 +0.000519 * DTL-88 +0.025560 * DTL-90 +0.014251 * DTL-93 +0.018469 * DTL-94 -0.019641 * DTL-96
