# Supplementary material for: Differentiation of acute and chronic vertebral compression fractures using conventional CT based on deep transfer learning features and hand-crafted radiomics features
Source: BMC Musculoskelet Disord. 2023 Mar 6;24:165. doi: 10.1186/s12891-023-06281-5 (PMC9987077; doi:10.1186/s12891-023-06281-5)
Supplement: Supplementary file 4 — Additional file 4. [file 12891_2023_6281_MOESM4_ESM.docx]

Deep Learning Radiomics feature importance score = 0.5973532523931404 + +0.155233 * DTL-0 -0.082287 * DTL-1 +0.040082 * DTL-2 -0.004683 * DTL-3 -0.054718 * DTL-5 +0.009508 * DTL-6 +0.000812 * DTL-7 +0.009286 * DTL-8 +0.023341 * DTL-10 +0.040776 * DTL-12 +0.012675 * DTL-13 -0.007443 * DTL-14 +0.001835 * DTL-15 +0.011944 * DTL-17 +0.011867 * DTL-18 -0.007822 * DTL-19 -0.022374 * DTL-20 +0.023604 * DTL-24 -0.018482 * DTL-27 -0.003819 * DTL-28 +0.010799 * DTL-29 +0.006050 * DTL-31 +0.005623 * DTL-38 -0.021528 * DTL-40 -0.028587 * DTL-43 -0.000099 * DTL-52 +0.009131 * DTL-56 -0.005914 * DTL-58 -0.002194 * DTL-62 +0.015927 * DTL-69 -0.006538 * DTL-70 +0.004250 * DTL-76 +0.024363 * DTL-77 -0.001474 * DTL-78 -0.006470 * DTL-79 +0.004326 * DTL-80 +0.003567 * DTL-82 -0.001076 * DTL-86 -0.027851 * DTL-90 +0.016614 * DTL-93 -0.000776 * DTL-94 -0.011956 * DTL-95 -0.011745 * DTL-97 -0.002585 * DTL-98 -0.024732 * exponential_glszm_SmallAreaHighGrayLevelEmphasis +0.007566 * log-sigma-1-0-mm-3D_firstorder_Median -0.009428 * log-sigma-3-0-mm-3D_firstorder_Skewness +0.037083 * log-sigma-3-0-mm-3D_glszm_ZoneVariance -0.088119 * log-sigma-5-0-mm-3D_firstorder_90Percentile +0.035348 * log-sigma-5-0-mm-3D_firstorder_Kurtosis +0.023971 * logarithm_firstorder_10Percentile +0.023261 * logarithm_firstorder_TotalEnergy +0.008087 * original_glcm_Idn +0.075978 * original_shape_Flatness +0.012433 * original_shape_Sphericity -0.042215 * original_shape_SurfaceVolumeRatio +0.027185 * square_glszm_SmallAreaLowGrayLevelEmphasis -0.034210 * squareroot_glcm_Correlation -0.014543 * wavelet-HHL_firstorder_Median -0.012381 * wavelet-HHL_glcm_ClusterShade -0.012291 * wavelet-HHL_glcm_Correlation +0.022335 * wavelet-HLH_firstorder_Median -0.020092 * wavelet-HLH_firstorder_Skewness +0.013014 * wavelet-HLH_glcm_Correlation +0.009402 * wavelet-HLL_firstorder_Mean -0.022997 * wavelet-HLL_firstorder_Skewness -0.002894 * wavelet-HLL_glcm_Correlation +0.005182 * wavelet-LHH_firstorder_Median -0.000204 * wavelet-LHH_glcm_Idn -0.022573 * wavelet-LHL_firstorder_Median -0.015160 * wavelet-LLH_firstorder_Median +0.007456 * wavelet-LLH_firstorder_Skewness +0.014177 * wavelet-LLH_glcm_ClusterShade +0.016521 * wavelet-LLH_glcm_Correlation
