# Supplementary material for: Differentiation of acute and chronic vertebral compression fractures using conventional CT based on deep transfer learning features and hand-crafted radiomics features
Source: BMC Musculoskelet Disord. 2023 Mar 6;24:165. doi: 10.1186/s12891-023-06281-5 (PMC9987077; doi:10.1186/s12891-023-06281-5)
Supplement: Supplementary file 5 — Additional file 5. [file 12891_2023_6281_MOESM5_ESM.docx]

Table 1. Diagnostic efficiency of different models with radiomics in the training and test cohorts.

|  | **model_name** | **Accuracy** | **AUC** | **95% CI** | **Sensitivity** | **Specificity** | **Threshold** | **F1-score** | **Task** |
| --- | --- | --- | --- | --- | --- | --- | --- | --- | --- |
| 0 | SVM | 0.923 | 0.973 | 0.955 - 0.990 | 0.952 | 0.920 | 0.668 | 0.950 | training cohort |
| 1 | SVM | 0.846 | 0.854 | 0.773 - 0.934 | 0.778 | 0.751 | 0.583 | 0.802 | test cohort |
| 2 | KNN | 0.826 | 0.934 | 0.913 - 0.955 | 0.837 | 0.902 | 0.800 | 0.881 | training cohort |
| 3 | KNN | 0.750 | 0.872 | 0.804 - 0.940 | 0.825 | 0.875 | 0.800 | 0.865 | test cohort |
| 4 | DecisionTree | 0.942 | 0.974 | 0.954 - 0.994 | 0.925 | 0.942 | 0.820 | 0.942 | training cohort |
| 5 | DecisionTree | 0.710 | 0.722 | 0.632 - 0.811 | 0.761 | 0.994 | 0.794 | 0.862 | test cohort |
| 6 | RandomForest | 0.995 | 0.999 | 0.999 - 1.000 | 0.992 | 0.949 | 0.600 | 0.979 | training cohort |
| 7 | RandomForest | 0.798 | 0.817 | 0.811 - 0.952 | 0.666 | 0.951 | 0.700 | 0.784 | test cohort |
| 8 | ExtraTrees | 0.858 | 0.874 | 0.854 - 0.994 | 0.994 | 0.894 | 0.850 | 0.963 | training cohort |
| 9 | ExtraTrees | 0.807 | 0.871 | 0.804 - 0.939 | 0.873 | 0.707 | 0.600 | 0.846 | test cohort |
| 10 | XGBoost | 0.934 | 0.964 | 0.954 - 0.994 | 0.905 | 0.954 | 0.907 | 0.935 | training cohort |
| 11 | XGBoost | 0.814 | 0.843 | 0.801 - 0.985 | 0.841 | 0.951 | 0.814 | 0.898 | test cohort |
| 12 | LightGBM | 0.844 | 0.899 | 0.852 - 0.984 | 0.994 | 0.994 | 0.622 | 0.995 | training cohort |
| 13 | LightGBM | 0.815 | 0.838 | 0.815 - 0.981 | 0.825 | 0.926 | 0.706 | 0.880 | test cohort |

Abbreviations: AUC, area under the curve; CI, confidence interval.

Table 2. Diagnostic efficiency of different models with DLR in the training and test cohorts.

|  | **model_name** | **Accuracy** | **AUC** | **95% CI** | **Sensitivity** | **Specificity** | **Threshold** | **F1-score** | **Task** |
| --- | --- | --- | --- | --- | --- | --- | --- | --- | --- |
| 0 | SVM | 0.949 | 0.992 | 0.993 - 0.999 | 0.944 | 0.961 | 0.668 | 0.958 | training cohort |
| 1 | SVM | 0.817 | 0.871 | 0.805 - 0.938 | 0.889 | 0.707 | 0.583 | 0.855 | test cohort |
| 2 | KNN | 0.841 | 0.912 | 0.886 - 0.938 | 0.857 | 0.817 | 0.600 | 0.867 | training cohort |
| 3 | KNN | 0.807 | 0.878 | 0.815 - 0.942 | 0.777 | 0.853 | 0.600 | 0.829 | test cohort |
| 4 | DecisionTree | 0.914 | 0.965 | 0.954 - 0.995 | 0.985 | 0.952 | 0.902 | 0.977 | training cohort |
| 5 | DecisionTree | 0.729 | 0.762 | 0.677 - 0.847 | 0.793 | 0.989 | 0.985 | 0.881 | test cohort |
| 6 | RandomForest | 0.922 | 0.998 | 0.996 - 1.000 | 0.992 | 0.993 | 0.600 | 0.993 | training cohort |
| 7 | RandomForest | 0.798 | 0.848 | 0.813 - 0.942 | 0.682 | 0.926 | 0.800 | 0.788 | test cohort |
| 8 | ExtraTrees | 0.916 | 0.976 | 0.945 - 1.000 | 0.990 | 0.990 | 0.990 | 0.991 | training cohort |
| 9 | ExtraTrees | 0.817 | 0.849 | 0.826 - 0.953 | 0.730 | 0.951 | 0.700 | 0.828 | test cohort |
| 10 | XGBoost | 0.885 | 0.974 | 0.964 - 0.999 | 0.975 | 0.926 | 0.939 | 0.963 | training cohort |
| 11 | XGBoost | 0.815 | 0.846 | 0.807 - 0.986 | 0.793 | 0.990 | 0.829 | 0.881 | test cohort |
| 12 | LightGBM | 0.894 | 0.914 | 0.905 - 1.000 | 0.994 | 0.925 | 0.821 | 0.973 | training cohort |
| 13 | LightGBM | 0.806 | 0.834 | 0.890 - 0.978 | 0.777 | 0.959 | 0.860 | 0.861 | test cohort |

Abbreviations: AUC, area under the curve; CI, confidence interval.

Table 3. Diagnostic efficiency of different models with Features Fusion in the training and test cohorts.

|  | **model_name** | **Accuracy** | **AUC** | **95% CI** | **Sensitivity** | **Specificity** | **Threshold** | **F1-score** | **Task** |
| --- | --- | --- | --- | --- | --- | --- | --- | --- | --- |
| 0 | SVM | 0.971 | 0.997 | 0.994 - 0.999 | 0.960 | 0.981 | 0.768 | 0.973 | training cohort |
| 1 | SVM | 0.885 | 0.915 | 0.855 - 0.974 | 0.936 | 0.829 | 0.683 | 0.914 | test cohort |
| 2 | KNN | 0.882 | 0.931 | 0.874 - 0.948 | 0.871 | 0.831 | 0.732 | 0.879 | training cohort |
| 3 | KNN | 0.857 | 0.881 | 0.875 - 0.931 | 0.798 | 0.841 | 0.769 | 0.839 | test cohort |
| 4 | DecisionTree | 0.954 | 0.975 | 0.941 - 0.994 | 0.921 | 0.945 | 0.825 | 0.941 | training cohort |
| 5 | DecisionTree | 0.769 | 0.792 | 0.715 - 0.821 | 0.802 | 0.978 | 0.828 | 0.883 | test cohort |
| 6 | RandomForest | 0.962 | 0.999 | 0.997 - 1.000 | 0.996 | 0.985 | 0.715 | 0.993 | training cohort |
| 7 | RandomForest | 0.852 | 0.862 | 0.801 - 0.882 | 0.785 | 0.926 | 0.700 | 0.856 | test cohort |
| 8 | ExtraTrees | 0.937 | 0.979 | 0.925 - 1.000 | 0.956 | 0.993 | 0.856 | 0.975 | training cohort |
| 9 | ExtraTrees | 0.854 | 0.863 | 0.812 - 0.911 | 0.831 | 0.963 | 0.725 | 0.895 | test cohort |
| 10 | XGBoost | 0.925 | 0.979 | 0.945 - 0.983 | 0.920 | 0.951 | 0.863 | 0.942 | training cohort |
| 11 | XGBoost | 0.851 | 0.846 | 0.811 - 0.932 | 0.806 | 0.985 | 0.865 | 0.887 | test cohort |
| 12 | LightGBM | 0.952 | 0.952 | 0.911 – 0.985 | 0.996 | 0.824 | 0.921 | 0.943 | training cohort |
| 13 | LightGBM | 0.844 | 0.896 | 0.814 - 0.932 | 0.815 | 0.931 | 0.827 | 0.876 | test cohort |

Abbreviations: AUC, area under the curve; CI, confidence interval.
